# Modeling and characterization of the electrical conductivity on metal nanoparticles/carbon nanotube/polymer composites

**DOI:** 10.1038/s41598-022-14596-x

**Published:** 2022-06-21

**Authors:** Yang Wang, Sijian Lu, Wenke He, Shen Gong, Yunqian Zhang, Xinsi Zhao, Yuanyuan Fu, Zhenghong Zhu

**Affiliations:** 1 School of Materials Science and Engineering, Central South University, Hunan, 410083 Changsha China; 2 School of Life Science, Central South University, Hunan, 410083 Changsha China; 3 State Key Laboratory of Powder Metallurgy, Changsha, 410083 China; 4 Department of Mechanical Engineering, York University, 4700 Keele Street, Toronto, ON M3J 1P3 Canada

**Keywords:** Materials science, Nanoscale materials, Structural properties

## Abstract

Flexible conductive films have good deformability and conductivity, and are expected to be used in flexible electronic devices. In this paper, four kinds of flexible conductive films were successfully prepared by compounding nano-sized metal (Ni, Cu, Au or AuCu alloy) particles to CNT surface and then dispersing to polydimethylsiloxane matrix. Experiment results show that the conductivity of these prepared films are almost two orders of magnitude higher than that of CNT/polydimethylsiloxane films with the same CNT loadings. A simulation model based on percolation network theory and Monte Carlo technology is introduced to study the influence of nanoparticles on the composite conductivity. Results confirmed that the introduction of nanoparticles effectively reduces the effective resistance of CNT and the tunnelling resistance at CNT junctions. The intrinsic conductivity and the length diameter ratio of CNT, the intrinsic conductivity, the size and the coverage ratio of nanoparticles are the core parameters affecting the conductivity of composite. Compared with CNT/polydimethylsiloxane films, the optimized theoretical conductivity of these nano-sized particles enhanced composites can be further improved.

## Introduction

Carbon nanotubes (CNTs) are widely used in electronic devices, sensors, actuators and biomaterials due to their excellent mechanical, electrical and thermal properties^1–9^. Many flexible conductive composite based on CNTs and elastic polymers, such as polydimethylsiloxane (PDMS), have been explored in depth^10^. The construction of CNT conductive network in almost insulated polymer matrix is the core of obtaining highly conductive flexible polymer composites. Increasing the CNT content can usually effectively improve the conductivity of polymer composites, however, it will inevitably reduce the flexibility of composites. Therefore, in order to maintain the flexibility of composites, the effective strategy is to increase the conductivity of CNT rather than increase the content of CNT.

To date, there have been several reports on improving the conductivity of CNT/polymer nanocomposites^11–18^. One of these various ways to improve the conductivity of the nanocomposite is to add metal particles into polymer matrix. For example, Cho used Au nanoparticles (NP) to prepare polystyrene/AuNP/CNT composite^19^. Zero-dimensional AuNP and one-dimensional CNT are dispersed together into the polystyrene matrix. And the conductivity of the composite is only 10^–4^ S/cm while the weight ratio of AuNP to polystyrene is 0.5. Typically, the intrinsic conductivity of CNTs is between 10^4^ S/m and 10^6^ S/m. Another effective method to improve the conductivity of CNT network is to compound highly conductive metal or alloy particles (such as Au and Cu, *σ*_Au_ = 4.16 × 10^7^ S/m and *σ*_Cu_ = 5.88 × 10^7^ S/m) on the surface of CNTs^20, 21^. However, Au is very expensive and is not conducive to large-scale applications. In contrast, Cu is much cheaper, but it is difficult to control the particle size (below 50 nm) and easy to oxidize. When synthesizing AuCu alloy, the amount of Au can be reduced as for the same coverage ratio. When synthesizing Ni nanoparticles on tubes, the particle size can be well controlled. And only enough metal particles are coated on the surface of tubes can ensure the high conductivity of CNT network.

In this paper, Ni, Cu, Au and AuCu alloy nanoparticles were composited on CNT surface. Then Metal/CNT was further dispersed into PDMS matrix to obtain a flexible Metal/CNT/PDMS composite film. The microstructure of the samples was characterized by scanning electron microscopy (SEM), X-ray diffraction (XRD), transmission electron microscopy (TEM) and scanning tunneling electron microscopy with high-angle annular dark field test (HAADF-STEM). Electrical conductivity of composites was measured by four-point method. Compared with CNT/PDMS flexible films, the conductivity of Metal/CNT/PDMS films is improved by nearly two orders of magnitude.

The introduction of metal particles not only improves the conductivity of CNT, but also affects the electron tunneling effect at the CNT junctions. Compared with pure CNT junctions, electrons need to enter the metal particles from the CNT at the metal/CNT junctions, and then reach another CNT by tunneling through the polymer insulating layer. In order to explore the influence of nano-sized metal particles on the conductivity of Metal/CNT/PDMS films, a simulation model based on percolation network (PN) theory and Monte Carlo (MC) technology is adopted. According to the experiment and simulation results, the core factors that affecting the conductivity of the Metal/CNT/polymer composite was discussed. In addition, the model also predicted the conductivity data of flexible composites by compounding different metal or alloy nanoparticles and adjusting various parameters of CNT network.

## Experimental

### Materials

Multi-wall CNT (The diameter ranges from 10 to 20 nm and the length ranges from 1 μm to 2 μm) is provided by Beijing Deco Island Gold Technology Co., Ltd. Polyethyleneimine is provided by Shanghai McLean Biochemical Technology Co., Ltd. (Shanghai, China). Chlorogluric Acid (> 47.8% in Au), Cupric Acetate Monohydrate, polyvinylpyrrolidone K30, sodium hypophosphite and ethylene glycol were provided by Chemical Reagents Co., Ltd. of Pharmaceutical Group.

### Metal/CNT Synthesis

First, 0.4 g purified CNT was mixed with chloroauric acid and Polyethyleneimine in a water bath at 60 °C for 1 h and followed by freeze-drying to prepare Au/CNT^22^. Copper ethylene glycol acetate solution (250 ml, 0.01 M) and sodium hypophosphite ethylene glycol solution (250 ml, 0.02 M) were mixed and 4.6 g PVP was added. The obtained Au/CNT was added to the above solution and ultrasound dispersion treatment was carried out until uniformity. After mixing, nitrogen is injected into the solution for 20 min at room temperature. Then it was moved into microwave heating equipment with a power of 700 w, a temperature of 60 °C and a reaction time of 5 min. The Au/Cu/CNT was obtained and dried in vacuum. Finally, AuCu/CNT was fully alloyed by reducing the particles in a tube furnace at 250 °C in 10% hydrogen and argon atmosphere for 8 h^23–26^. 20 mg of purified CNTs was stirred with 0.005 M of Ni(NO_3_)_2_·6H_2_O and 1.4 g of PVP in water solution (40 ml). Then 40 ml of 0.015 M NaBH_4_ solution was dropped into the mixed solution under ice bath for 10 h. Finally, the precursor was filtered and then reduced at 400 °C in a hydrogen-argon atmosphere for 2 h to obtain Ni/CNT. 0.1 g purified CNT was dispersed in 0.1 M SnCl_2_/0.1 M HCl aqueous solution for 30 min and then filtered and washed with 0.014 M PdCl_2_/0.25 M HCl aqueous solution and deionized water. 0.006 mol CuSO_4_.5H_2_O, 0.013 mol C_2_H_2_O_3_, 0.011 mol EDTANa_2_ and 1 g PVP was added in 100 ml deionized water with 0.1 g purified CNT. The pH, temperature and time of electroless copper plating bath was set as 12.5, 283 K and 5 h, respectively. Cu/CNT was prepared by filtering and washing with deionized water.

### Metal/CNT/PDMS Synthesis

Firstly, PDMS and its curing agent were mixed uniformly in a ratio of 10:1 to prepare PDMS substrate with a thickness of 3 mm and a radius of 5 cm^27, 28^. Then the prepared Ni/CNT, Cu/CNT, Au/CNT and AuCu/CNT were dissolved in toluene (TL) respectively. After ultrasound dispersion, they were added into PDMS/curing agent mixture. After vacuum defoaming, Metal/CNT/PDMS was spin-coated on the prefabricated PDMS substrates with various speed to obtain the same film thickness (120 μm ± 5 um). Finally, the prepared Metal/CNT/PDMS samples were vacuum dried for 24 h at 40 °C.

### Properties characterization and simulation procedure

Four-point probe method (Keithley 2450) was used to measure the conductivity of the composite films. In order to avoid the changes of contact resistance on films, probes with an equal spacing of 1 mm were used. The experimental data of each state are obtained by averaging 50 position points in five samples. All Metal/CNT samples are also expressed uniformly by the CNT loading.

According to PN theory, a certain amount of CNT will form a conductive network in the polymer matrix, and its resistance value should consider all the resistance components in the network^29, 30^. By using a MC simulation, the total resistance of the network can be calculated^31–34^. More details are shown in supporting information. The following two assumptions are used in the modeling process. First, the metal or alloy nanoparticles are evenly dispersed on the surface of the tubes. Second, the tubes are well dispersed in the polymer matrix.

## Results and discussion

### Preparation of the composite

**Figure 1:**
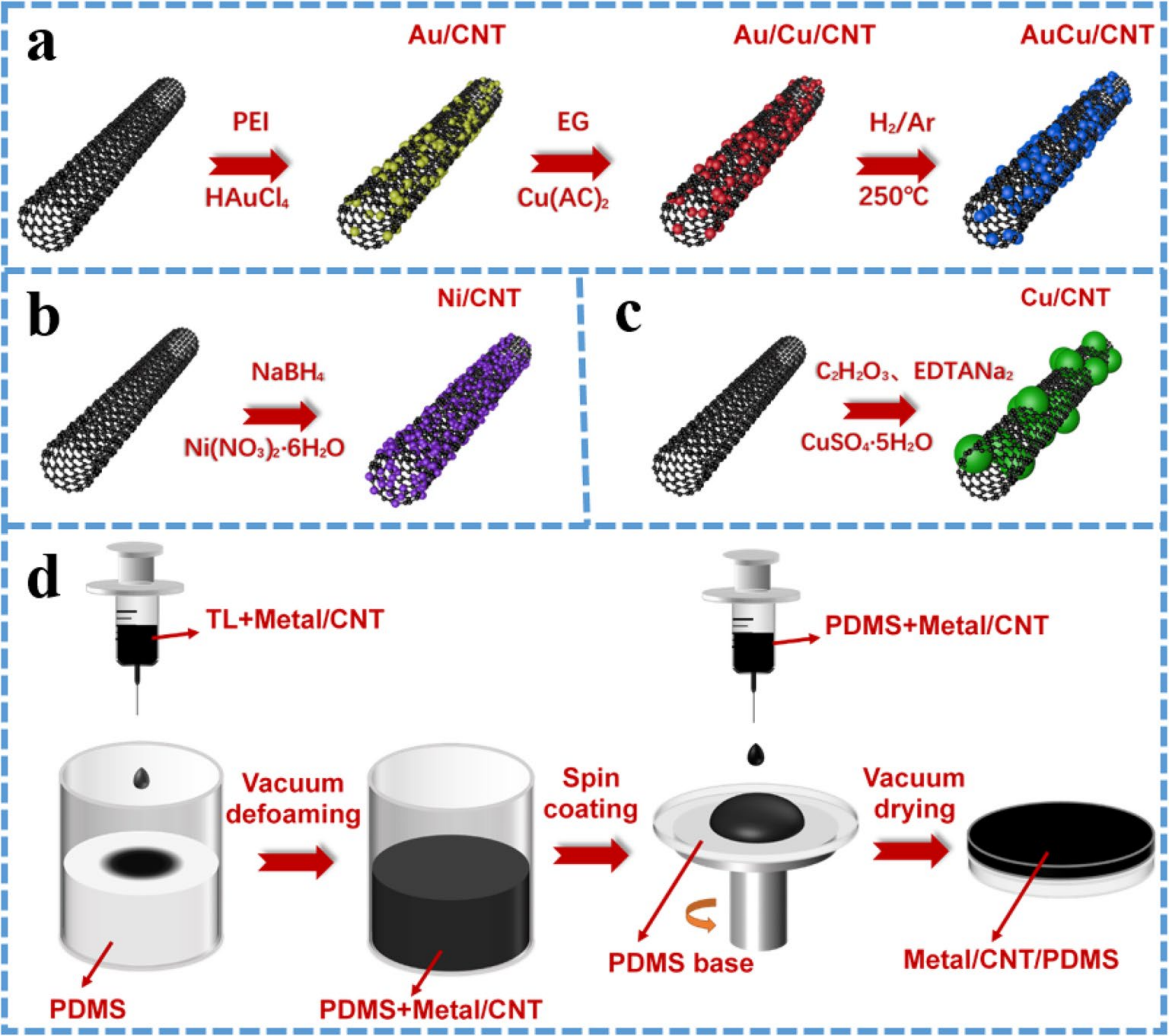
Schematic diagram of (**a**) Au/CNT and AuCu/CNT preparation process, (**b**) Ni/CNT preparation process, (**c**) Cu/CNT preparation process and (**d**) Metal/CNT/PDMS films preparation process.

Figure 1a shows the schematic diagram of the preparation process of Au/CNT and AuCu/CNT. In order to ensure the uniform dispersion of Au particles on tubes, PEI is adsorbed on the purified tubes by electrostatic interaction^22^. The defects on carbon tubes become the nucleation point of Au. By reducing the chloraurate radical, the uniformly adhered Au particles are obtained. In the generated Au/CNT samples, Au occupied almost all defect point. Au/Cu/CNT is obtained on the basis of Au/CNT. After adding Cu salt, the original Au particles on CNT are used as the nucleation point of Cu. Some Cu ions entered the interior of Au particles (but the alloying is incomplete, and some are in a disordered state), and the other part nucleated and grew around Au particles (or even cover the surface of Au particles). And then AuCu/CNT is prepared by further reduction and alloying with hydrogen and argon^35^.

Figure 1b and c shows the schematic diagram of the Ni/CNT and Cu/CNT preparation process, respectively. Metal particles with different morphologies were obtained by different Metal/CNT preparation processes. Figure 1d shows the schematic diagram of Metal/CNT/PDMS film preparation process. After mixing Metal/CNT with PDMS uniformly, the film thickness was controlled by spin coating on pre-prepared substrates. CNT/PDMS and Metal/CNT/PDMS films were prepared by the same method.

### Microstructure characterization of raw materials

**Figure 2:**
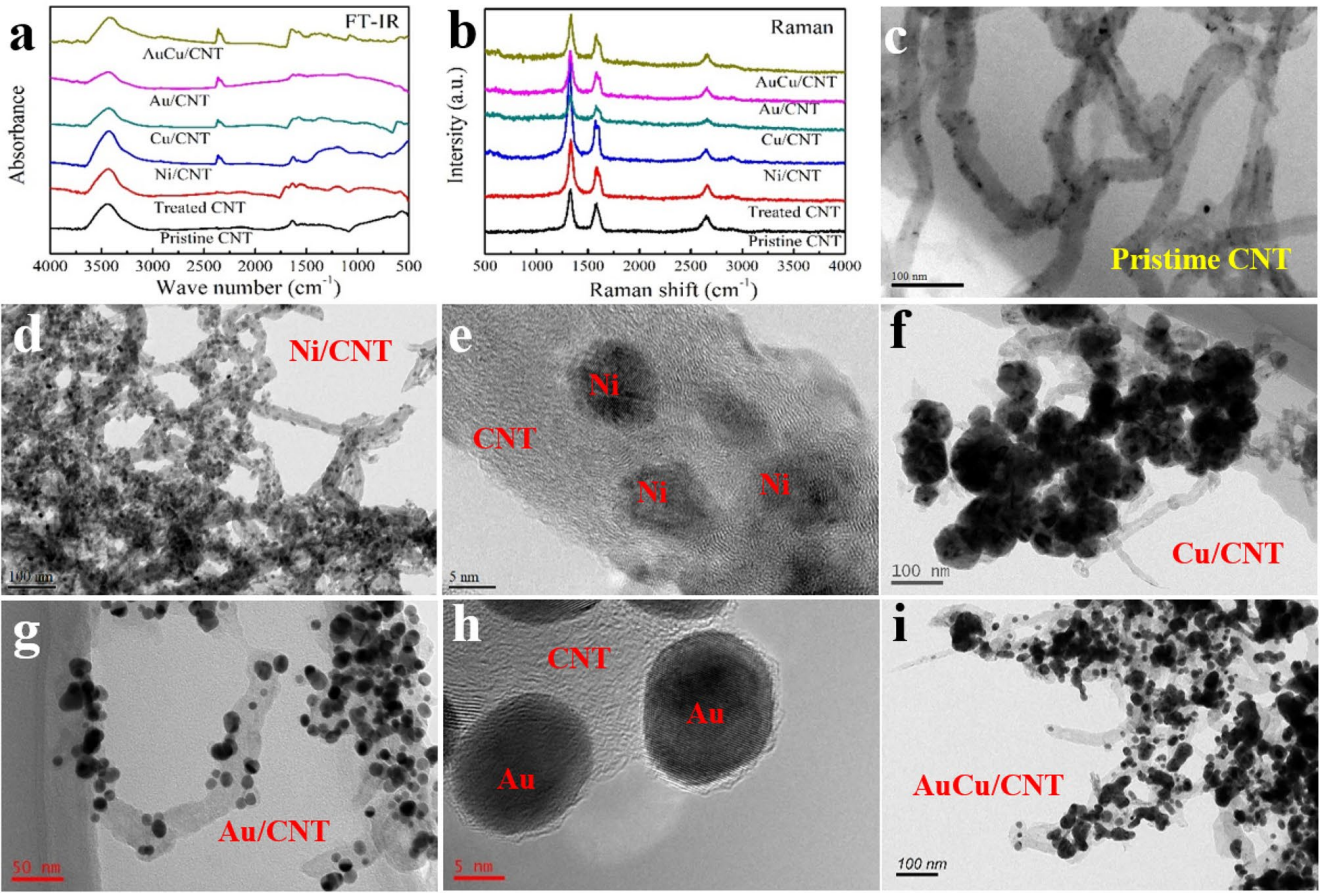
(**a**) FT-IR results of pristine CNT, purified CNT, Ni/CNT, Cu/CNT, Au/CNT and AuCu/CNT. (**b**) Raman results of pristine CNT, purified CNT, Ni/CNT, Cu/CNT, Au/CNT and AuCu/CNT. Morphology of (**c**) Purified CNT, (**d**, **e**) Ni/CNT, (**f**) Cu/CNT, (**g**, **h**) Au/CNT, (**i**) AuCu/CNT.

As shown in Fig. 2a and b, Fourier transform infrared (FT-IR) spectra and Raman test was used on the samples of pristine CNT, purified CNT, Ni/CNT, Cu/CNT, Au/CNT and AuCu/CNT. The functional groups present in samples are analysed by recording FT-IR spectrum in the wavenumber range of 500–4000 cm^−1^. The oscillation of the carboxyl group on the surface of the carbon nanotube causes the O–H stretch vibration of the hydroxyl group to appear a wide valley at 3450 cm^−1^. Other exhibited representative peaks at 1730, 1627, 1394, 1245 and 1090 cm^−1^, corresponding to C = O stretch, aromatic C = C and O–H bending, C–OH groups, epoxy C–O stretch, and alkoxy C–O stretch, respectively. Compared with pristine CNTs, characteristic peaks of C = O and C–O structures were observed on treated tubes, indicating the implantation of COOH groups on the surface. That band 2380, 1627 and 1394 cm^-1^ increased to some degree after the metal and alloy particles coated on tubes, indicating that possible interactions occurred between OH and COOH functional groups with the metal and alloy particles. As can be seen in Fig. 2b (Raman spectra), a D band at 1350 cm^–1^ and a G band at 1580 cm^-1^ were observed in all samples. According to the enhancement of D band, more defects exist on the treated tubes than pristine ones.

Figure 2c–i shows the morphology of purified CNT, Ni/CNT, Cu/CNT, Au/CNT and AuCu/CNT powders, respectively. As shown in figures, a lot of small-sized metal particles are coated on tubes and the average diameter of the tubes is 15 nm, while the average size of Ni, Cu, Au and AuCu particles are about 10 nm, 100 nm, 20 nm and 30 nm, respectively. Metal particles are basically spherical and evenly distributed on CNT, and the Ni/CNT has the highest coverage ratio on the surface of tubes. The preparation process of AuCu alloy is based on Au/CNT. By microwave heating of copper ethylene glycol acetate solution containing sodium hypophosphite, Cu particles can be obtained on the surface and adjacent positions of Au particles. During the following alloying process, Au and Cu fuse to form spherical particles with rounded surface.

**Figure 3:**
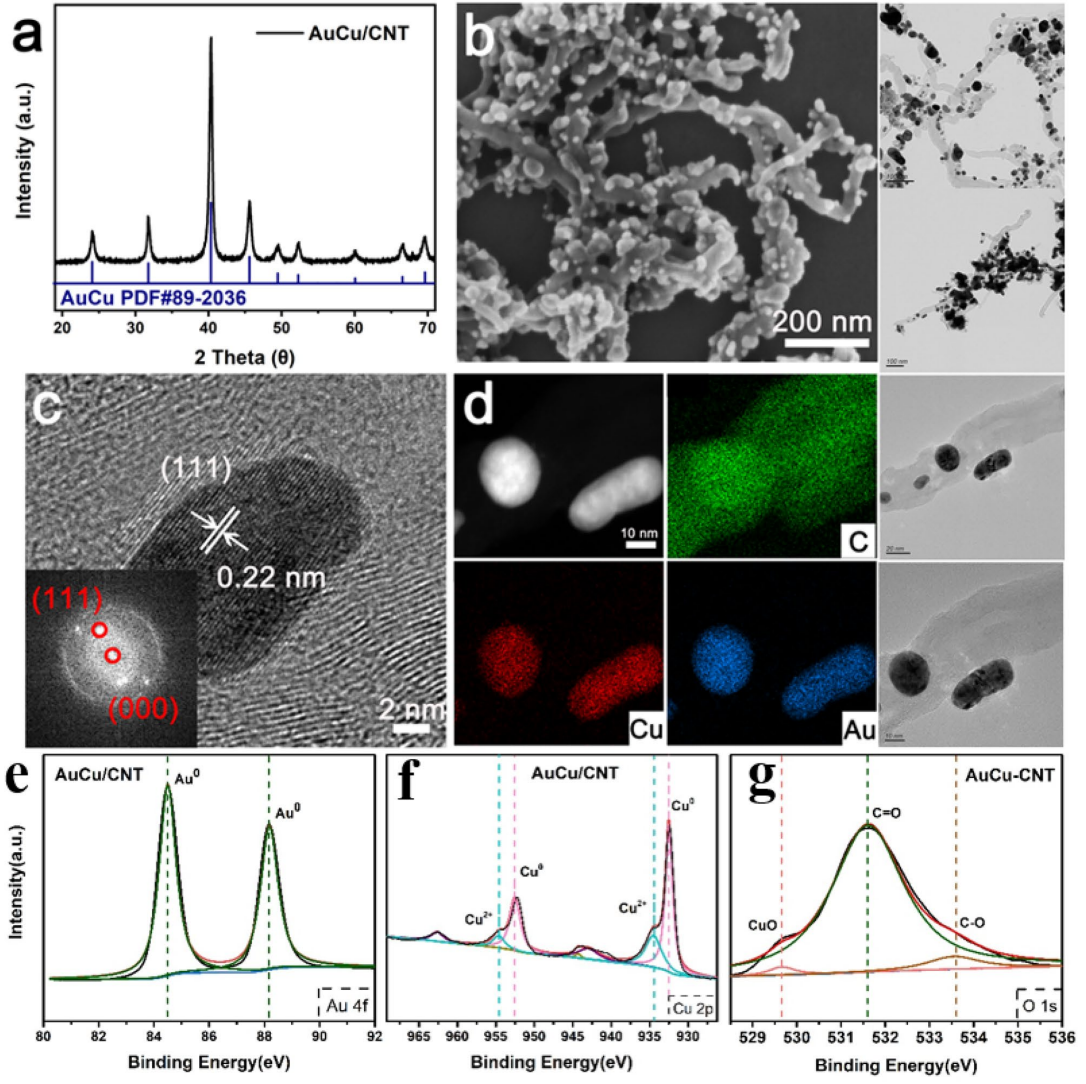
(**a**) XRD spectra of AuCu/CNT, (**b**) Microstructure of AuCu/CNT, (**c**) High Resolution Morphology of AuCu/CNT (The illustration is the fast Fourier transform image of selective diffraction), (**d**) HAADF-STEM image of AuCu/CNT, (**e**) Au 4f. XPS spectra of AuCu/CNT, (**f**) Cu 2p XPS spectra of AuCu/CNT, (**g**) O 1 s XPS spectra of AuCu/CNT.

Next, more details of AuCu/CNT are shown in Fig. 3. The XRD diffraction pattern (Fig. 3a) of AuCu/CNT is identical with the peak position of AuCu^23^. The sharp diffraction peaks indicate that AuCu particles in AuCu/CNT have the same structure as AuCu crystals and belong to face-centered cubic structure. It is worth mentioning that XRD results show that there is no diffraction peak corresponding to Au or Cu in AuCu/CNT powders. This indicates that only AuCu nano-alloy remains on the CNT surface during the preparation process. Figure 3b shows the SEM morphology of CNT coated with AuCu alloy. Scanning images show that AuCu particles are still uniformly coated in a wider field of vision. Figure 3c shows a high resolution image of nano-scale AuCu alloy particles loaded on tubes. It can be seen from the figure that the high resolution of the whole AuCu alloy nanoparticles is composed of a group of parallel lattice fringes with a spacing of 0.22 nm, which is consistent with the (111) plane spacing of AuCu alloy^23^. The illustration is a diffraction spot of the corresponding region after fast Fourier transform.

Figure 3d shows the distribution of elements in AuCu alloy. It shows that the content of Au and Cu elements in particles is equal and uniform, which is consistent with the XRD results. Figure 3e–g show the XPS data of as-prepared AuCu/CNT samples. The Au 4f. spectra (Fig. 3e) indicate that Au in AuCu/CNT samples was in metallic state. The Cu 2p spectra (Fig. 3f) of AuCu/CNT samples exhibit Cu 2p3/2 and 2p1/2 doublet peaks centered at ∼932.5 and ∼952.5 eV, indicating that most of Cu was kept as AuCu alloy in the samples, consistent with our XRD and HAADF-STEM results. According to Fig. 3, the coated particles on the surface of CNT are alloyed AuCu particles with equal atomic ratio.

### Microstructure characterization of the composite

**Figure 4:**
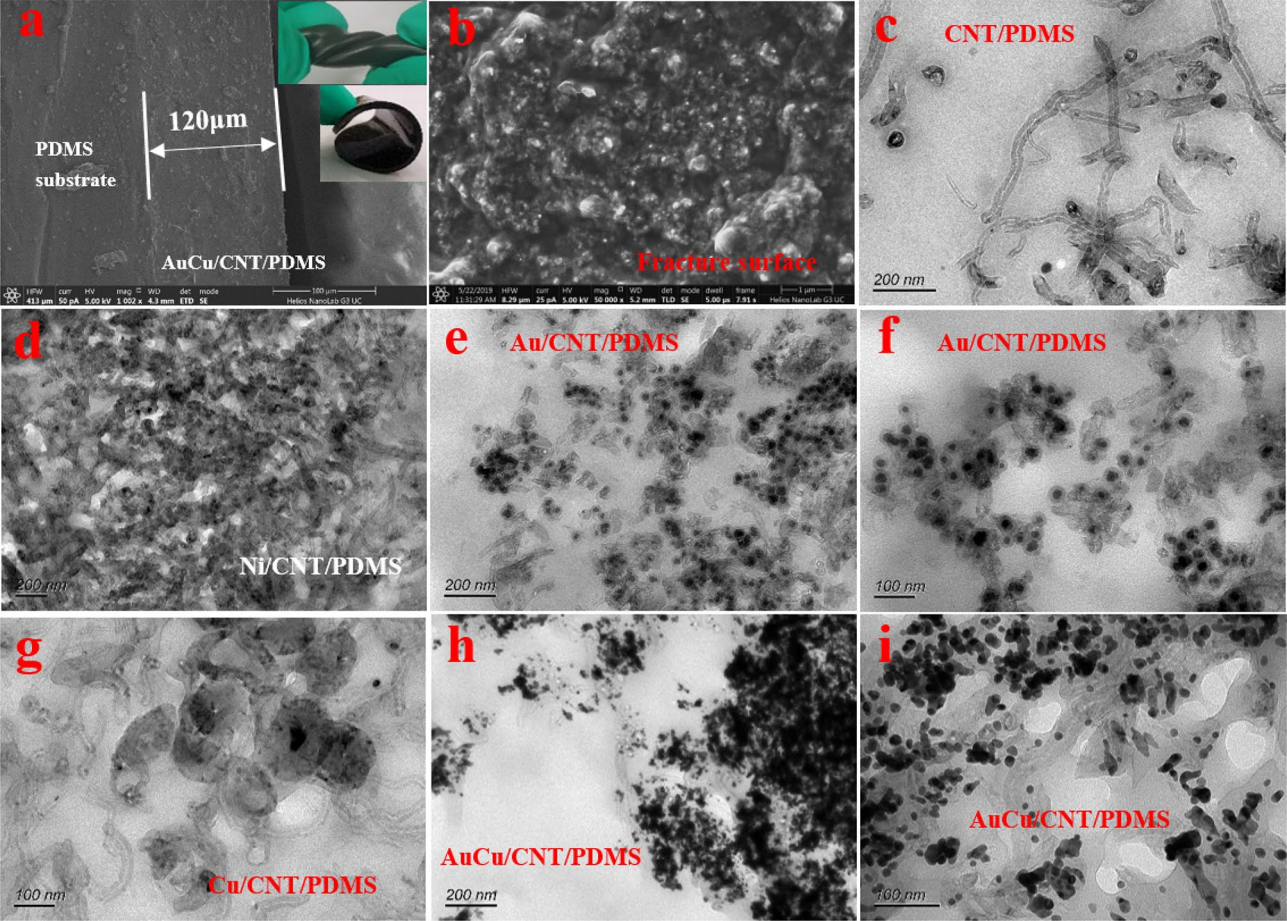
SEM morphology of (**a**) Cross section and (**b**) Fracture surface of AuCu/CNT/PDMS films. TEM morphology of (**c**) CNT/PDMS samples, (**d**) Ni/CNT/PDMS samples, (**e**, **f**) Au/CNT/PDMS samples, (**g**) Cu/CNT/PDMS samples, (**h**, **i**) AuCu/CNT/PDMS samples.

Then, the microstructure of the composites was characterized. Figure 4a shows the cross section of AuCu/CNT/PDMS films and inserted picture shows macroscopic photographs of the flexible films. Conductive film is uniformly spin-coated on PDMS substrate and the thickness of AuCu/CNT/PDMS film is about 120 μm. Figure 4b shows the fracture surface of AuCu/CNT/PDMS films with 8 wt.% CNT loadings. As can be seen from the figure, tubes were dispersed uniformly on the fracture surface. Figure 4c–i presents the TEM morphology of CNT/PDMS, Ni/CNT/PDMS, Au/CNT/PDMS, Cu/CNT/PDMS and AuCu/CNT/PDMS samples, respectively. As shown in the figures, CNT and Metal/CNT are evenly distributed in the PDMS matrix. Metal/CNT is well combined with polymer matrix without bubble and other defects. Compared with the Metal/CNT powder morphology in Fig. 2, the size and morphology of metal nanoparticles loaded on CNT are basically consistent with the original powder sample. In addition, the metal particles in the flexible film are still closely combined with tubes without obvious falling off phenomenon. The above indicate that the preparation process of flexible films did not change the size, morphology and distribution of metal nanoparticles.

### Calculation and simulation

In order to further explore the influence of nano-sized metal particles on the conductivity of Metal/CNT/PDMS films, computer simulation based on PN theory and MC simulations is introduced. According to the previous research of our group, the electrical properties of CNT/PDMS films is calculated based on the intrinsic resistance of tubes segment and the contact resistance between tubes^34^. The tubes that without conduction current is eliminated by dulmage-mendelsohn decomposition. The intrinsic resistance *R*_*int*_ along tubes between two nearest contact points *j* and *k* can be evaluated as:

1 $$R_{{\text{int}}} = \frac{{4l_{{{\text{jk}}}} }}{{\pi \left( {\sigma_{CNT} + \delta_{Metal} \sigma_{Metal} } \right)D_{CNT}^{{2}} }}$$

where *l*_*jk*_ is the length of the tubes segment between the points* j* and *k*, *δ*_*Metal*_ is the coverage ratio of metal and alloy particles on the tubes. *D*_*CNT*_ is the tube’s diameter, σ_*CNT*_ is the intrinsic electrical conductivity of tubes, σ_*Metal*_ is the metal particle’s intrinsic electrical conductivity, respectively.

Another resistance component, the contact resistance *R*_*c*_, is evaluated by Landauer-Büttiker (LB) formula as:

2 $$R_{{\text{c}}} \approx \frac{h}{{2e^{2} }} \cdot \frac{1}{{M{\mathcal{T}}}}$$

where *e* is the electron charge, *h* is Planck’s constant and *M* is the channel number. *T* is the transmission probability, which can be calculated as:

3 $${\mathcal{T}}{ = }\left\{ {\begin{array}{*{20}l} {\exp \left( {\frac{{ - d_{vdw} }}{{{h \mathord{\left/ {\vphantom {h {\sqrt {8m_{e} \left| {W_{Metal} - W_{CNT} } \right|} }}} \right. \kern-\nulldelimiterspace} {\sqrt {8m_{e} \left| {W_{Metal} - W_{CNT} } \right|} }}}}} \right)} \hfill & {0 < d \le D_{CNT} + D_{{{\text{M}}etal}} + 2d_{vdw} } \hfill \\ {\exp \left( {\frac{{ - \left( {d - D_{CNT} - D_{Metal} - d_{vdw} } \right)}}{{{h \mathord{\left/ {\vphantom {h {\sqrt {8m_{e} \left| {W_{Metal} - W_{PDMS} } \right|} }}} \right. \kern-\nulldelimiterspace} {\sqrt {8m_{e} \left| {W_{Metal} - W_{PDMS} } \right|} }}}} + \frac{{ - d_{vdw} }}{{{h \mathord{\left/ {\vphantom {h {\sqrt {8m_{e} \left| {W_{Metal} - W_{CNT} } \right|} }}} \right. \kern-\nulldelimiterspace} {\sqrt {8m_{e} \left| {W_{Metal} - W_{CNT} } \right|} }}}}} \right)} \hfill & {D_{CNT} + D_{Metal} + 2d_{vdw} < d \le D_{CNT} + 2D_{Metal} + 3d_{vdw} } \hfill \\ {\exp \left( {\frac{{ - \left( {d - D_{CNT} - 2D_{Metal} - 2d_{vdw} } \right)}}{{{h \mathord{\left/ {\vphantom {h {\sqrt {8m_{e} \left| {W_{Metal} - W_{PDMS} } \right|} }}} \right. \kern-\nulldelimiterspace} {\sqrt {8m_{e} \left| {W_{Metal} - W_{PDMS} } \right|} }}}} + \frac{{ - 2d_{vdw} }}{{{h \mathord{\left/ {\vphantom {h {\sqrt {8m_{e} \left| {W_{Metal} - W_{CNT} } \right|} }}} \right. \kern-\nulldelimiterspace} {\sqrt {8m_{e} \left| {W_{Metal} - W_{CNT} } \right|} }}}}} \right)} \hfill & {D_{CNT} + 2D_{Metal} + 3d_{vdw} < d \le d_{cutoff} } \hfill \\ \end{array} } \right.$$

where *m*_*e*_ is the electron mass, *d* is the distance between two axes of tubes, *W*_*CNT*_*, W*_*Metal*_ and *W*_*PDMS*_ is the work functions of tubes, metal particles and PDMS matrix, respectively. *d*_*vdw*_ and *d*_*cutoff*_ is the Van der Waals distance and maximum effective tunneling distance, respectively. Table 1 shows some experimental observation data and model input parameters of Metal/CNT/PDMS films, which is used to calculate the conductivity of Metal/CNT/PDMS films.

**Table 1:** Experimental data and model input parameters of Metal/CNT/PDMS composites.

Items	CNT	Ni/CNT	Cu/CNT	Au/CNT	AuCu/CNT
*L*_*CNT*_ (μm)	1–2	1–2	1–2	1–2	1–2
*D*_*CNT*_ (nm)	10–20	10–20	10–20	10–20	10–20
*σ*_*CNT*_ (S/m)	10^4^	10^4^	10^4^	10^4^	10^4^
*σ*_*Metal*_ (S/m)	/	1.28 × 10^7^	5.88 × 10^7^	4.16 × 10^7^	4.82 × 10^7^
*D*_*Metal*_ (nm)	/	5–20	30–150	5–30	5–40
*δ*_*Metal*_ (%)	/	80	60	60	70
*W*_*CNT*_ (eV)	4.70	4.70	4.70	4.70	4.70
*W*_*Metal*_ (eV)	/	5.00	4.65	5.10	4.85
*W*_*PDMS*_ (eV)	4.00	4.00	4.00	4.00	4.00

**Figure 5:**
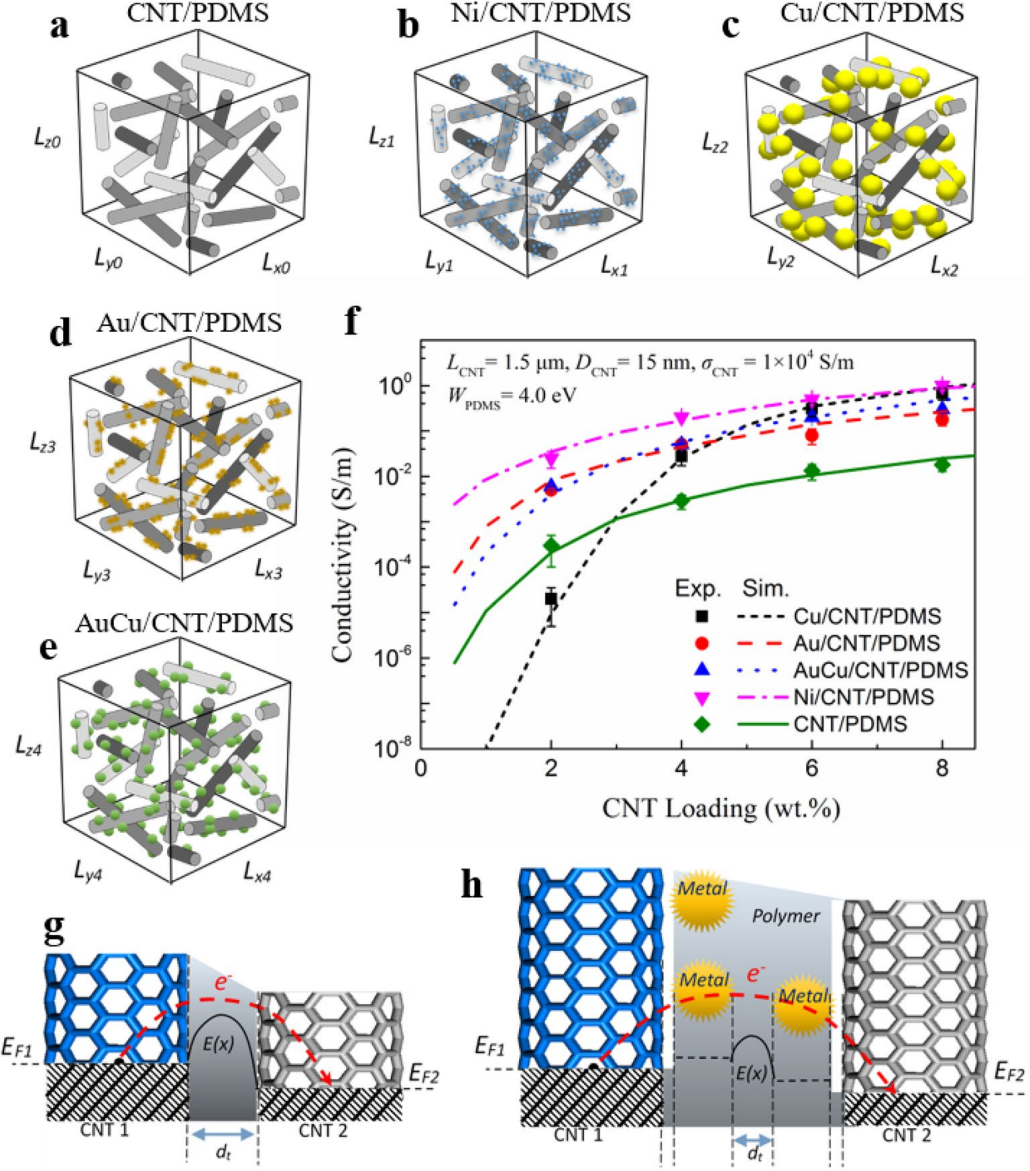
Schematic diagram of RVC on (**a**) CNT/PDMS, (**b**) Ni/CNT/PDMS, (**c**) Cu/CNT/PDMS, (**d**) Au/CNT/PDMS, (**e**) AuCu/CNT/PDMS. Comparison of experiment and simulation data on Metal/CNT/PDMS films (**f**). Schematic diagram of (**g**) CNT junctions and (**h**) Metal/CNT junctions.

Figure 5 shows the schematic diagram of RVC (representative volume cuboid, 5 mm × 5 mm × 5 mm), comparison of experimental and simulation data and the schematic diagram of conducting mechanism on junctions. Figure 5a–e shows the RVC which represents CNT, Ni/CNT, Cu/CNT, Au/CNT and AuCu/CNT in PDMS matrix, respectively. As enough tubes dispersed into polymer matrix, tunneling junctions formed between them. Then the tubes network is constructed in the non-conductive polymer matrix and thus conductive composites are realized. Figure 5f presents the experiment and simulation data of Ni/CNT/PDMS, Cu/CNT/PDMS, Au/CNT/PDMS and AuCu/CNT/PDMS, respectively. As shown in the figure, the simulation results are very close to the experimental data. With the increase of CNT and Metal/CNT content, the conductivity of PDMS films increases rapidly. The growth of different metal particles on CNT will greatly affect the conductivity of CNT/PDMS films. Compared with pure CNT/PDMS films, the introduction of uniform metal nanoparticles can greatly (1–2 orders of magnitude) improve the conductivity of the flexible composite films. Only Cu/CNT/PDMS composite films with 2wt.% CNT content have lower conductivity than CNT/PDMS, which may be related to the relatively large size of metal Cu particles. And this will be discussed later.

As shown in Fig. 5g, electrons cross the polymer barrier to another CNT through quantum tunneling effect at the CNT junctions. While at the Metal/CNT junctions (see Fig. 5h), electrons are first transmitted from CNT to metal particles, and then tunneled from metal particles across the polymer barrier to another CNT or metal particles on CNT. In this process, the intrinsic conductivity, size, work function and other parameters of metal particles will affect the tunneling process, and ultimately affect the contact resistance in the conductive network. It is worth mentioning that the metal or alloy particles coated on tubes will also directly affect the equivalent conductivity of CNTs.

**Figure 6:**
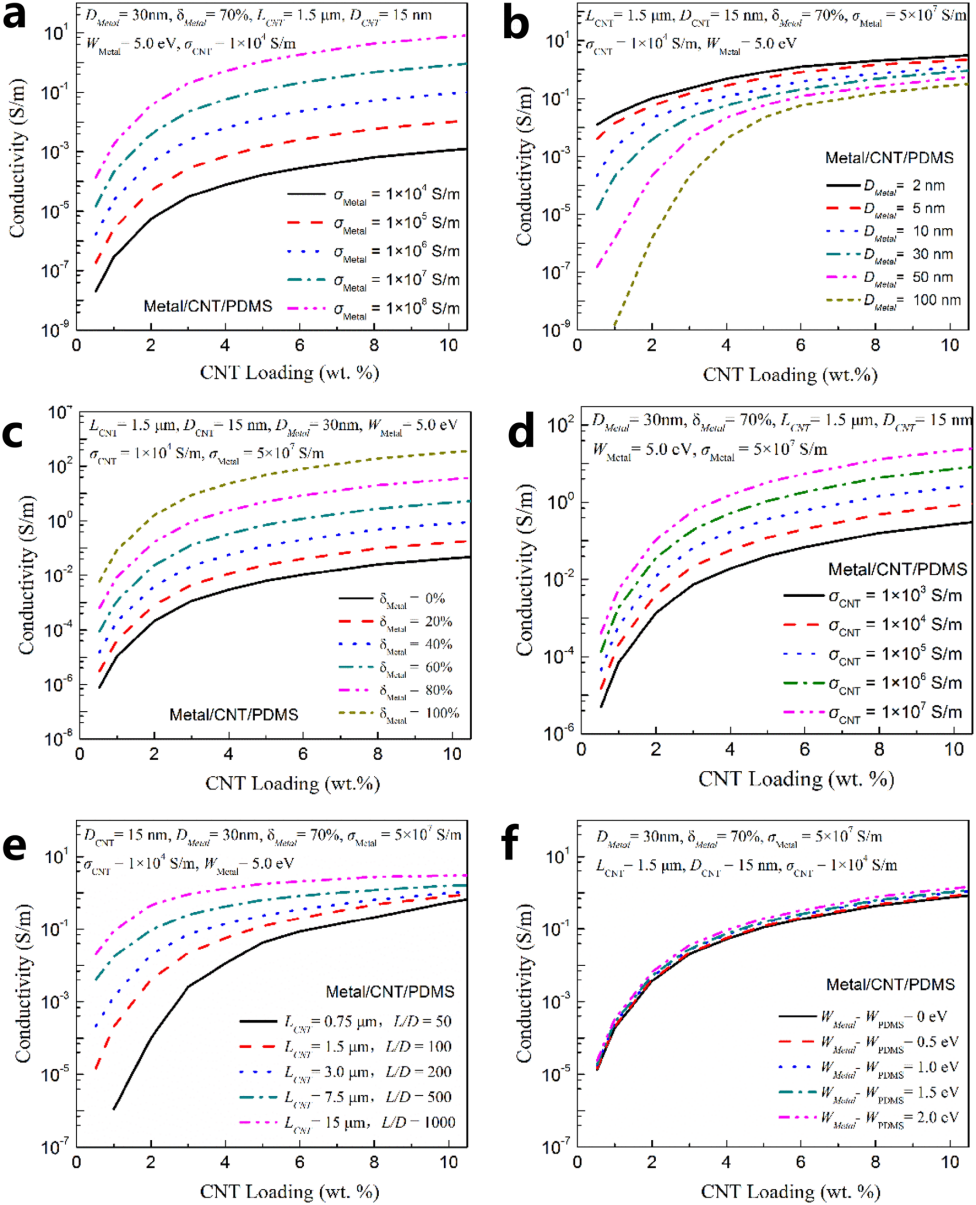
Calculation and simulation results of conductivity on Metal/CNT/PDMS films with various (**a**) *σ*_*Metal*_, (**b**) *D*_*Metal*_, (**c**) *δ*_*Metal*_, (**d**) *σ*_*CNT*_, (**e**) *L*_*CNT*_, (**f**) *W*_*Metal*_*-W*_*PDMS*_.

The conductivity of Metal/CNT/polymer films is related to many factors, such as the intrinsic conductivity of CNT, the length diameter ratio of tubes, the intrinsic conductivity of metal particles, the size of metal particles, the coverage ratio of metal particles, the metal’s work function, the polymer’s work function and so on. On the basis of successfully fitting the data of four prepared Metal/CNT/polymer films, these influencing factors are calculated and analyzed one by one. Figure 6 shows the calculation and simulation results of conductivity on Metal/CNT/PDMS films with various *σ*_*Metal*_, *D*_*Metal*_, *δ*_*Metal*_, *σ*_*CNT*_, *L*_*CNT*_ and *W*_*Metal*_*-W*_*PDMS*_. As shown in the figure, among the above parameters, the intrinsic conductivity of CNT and metal particles and the coverage ratio of metal particles have similar effects on the conductivity of composite films. That is, the increase of these parameters will significantly reduce the resistance of the CNT conductive section in the conductive network. Therefore, the film’s conductivity enhanced rapidly with the increase of these three parameters.

In addition, the length diameter ratio of CNT and the diameter of metal particles have similar effects on the conductivity of composite films. That is, too small length diameter ratio of CNT and too large metal particles will lead to a significant decrease in the conductivity of composite films in the case of low CNT loadings. This may be directly related to the difficulty of CNT and Metal/CNT forming an effective conductive network in the polymer matrix. The difference of work function between metal and polymer has limited influence on the conductivity of composites. In general, the greater the difference, the higher the conductivity of composites.

**Figure 7:**
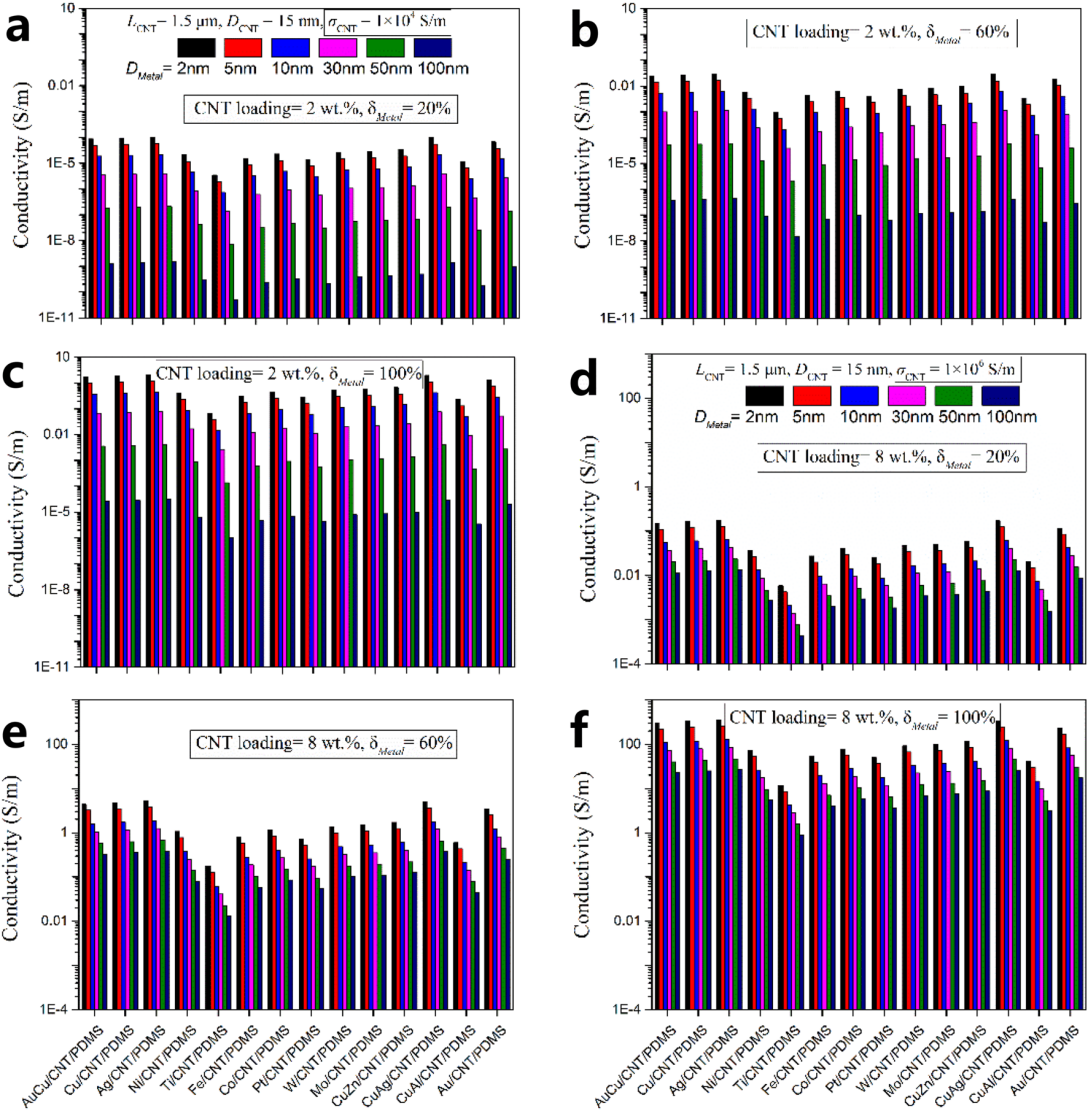
Calculation and simulation results of conductivity on Metal/CNT/PDMS films with various metal and alloys coated on tubes, various CNT loadings, various metal coverage ratio and various intrinsic conductivity of CNT. (**a**) *σ*_*cnt*_ = 10^4^ S/m, CNT loading = 2 wt.%, *δ*_*Metal*_ = 20%. (**b**) *σ*_*cnt*_ = 10^4^ S/m, CNT loading = 2 wt.%, *δ*_*Metal*_ = 60%. (**c**) *σ*_*cnt*_ = 10^4^ S/m, CNT loading = 2 wt.%, *δ*_*Metal*_ = 100%. (**d**) *σ*_*cnt*_ = 10^6^ S/m, CNT loading = 8 wt.%, *δ*_*Metal*_ = 20%. (**e**) *σ*_*cnt*_ = 10^6^ S/m, CNT loading = 8 wt.%, *δ*_*Metal*_ = 60%. (**f**) *σ*_*cnt*_ = 10^6^ S/m, CNT loading = 8 wt.%, *δ*_*Metal*_ = 100%.

In order to obtain high conductivity flexible composite films, it is very important to reasonably select the core parameters such as metal particle type, metal particle size and coverage ratio, original CNT and Metal/CNT loadings. Figure 7 shows the calculation and simulation results of conductivity on Metal/CNT/PDMS films with various metal and alloys coated on tubes, various CNT loadings, various metal coverage ratio and various intrinsic conductivity of CNT. Specifically, the intrinsic conductivity of tubes is between 10^4^ and 10^6^ S/m. The higher the conductivity, the more expensive it will be. The simulation data of Fig. 7a–c are calculated on the basis that the intrinsic conductivity of CNT is set as 10^4^ S/m and CNT loading is set as 2 wt.%, while that of Fig. 7d–f are calculated on the basis of 10^6^ S/m and 8 wt.%. The calculation results show that among various metals and alloys, Ag/CNT, CuAg/CNT, Cu/CNT, AuCu/CNT and Au/CNT have obvious advantages. In these systems, when the CNT content is 8 wt.%, the metal particles coverage ratio is 100% and the tube’s intrinsic conductivity is 10^6^ S/m, the flexible film’s conductivity can reach more than 100 S/m.

On the premise of the same Metal/CNT loading, reducing metal particle size and increasing coverage ratio are effective means to greatly enhance the film’s conductivity. Further increasing the Metal/CNT loading can increase the film’s conductivity, but it will also weaken the flexibility of the film. In addition, tubes with larger aspect ratio are more prone to agglomeration, which will seriously affect the metal particle composite process and subsequent polymer composite process. The composite process of different metals or alloys on CNT will greatly affect the size and coverage ratio of metal particles, therefore, the selection of Metal/CNT preparation process is also very important.

## Conclusion

Ni/CNT, Cu/CNT, Au/CNT and AuCu/CNT were successfully obtained by compounding uniform dispersed nano-sized metal particles on CNT surface. XRD and HAADF-STEM results suggested that alloyed AuCu particles with equal atomic ratio were combined on the tube surface. On this basis, the corresponding conductive flexible films on PDMS matrix with various CNT loadings were prepared. Electrical conductivity test results shown that most of the prepared Metal/CNT/PDMS films are more conductive than CNT/PDMS films. After experimental verification, a simulation model based on percolation network theory and Monte Carlo technology is adopted to further explore the influence of nano-sized metal and alloy particles on the film’s conductivity. Simulation results confirmed that the intrinsic conductivity and the aspect ratio of tubes, the intrinsic conductivity, the size and the coverage ratio of metal particles are the core factors affecting the Metal/CNT/polymer film’s conductivity. Calculation results also indicate that the conductivity of flexible composites can reach more than 100 S/m in Ag/CNT/PDMS, CuAg/CNT/PDMS, Cu/CNT/PDMS, AuCu/CNT/PDMS or Au/CNT/PDMS films, as the CNT loading is 8 wt.%, the metal particles coverage ratio is 100% and the intrinsic conductivity of CNT reaches 10^6^ S/m.

## Supplementary Information

Supplementary Information.

## Data Availability

The datasets used and/or analyzed during the current study are available from the corresponding author on reasonable request.
