# Supplementary material for: Modeling and characterization of the electrical conductivity on metal nanoparticles/carbon nanotube/polymer composites
Source: Sci Rep. 2022 Jun 21;12:10448. doi: 10.1038/s41598-022-14596-x (PMC9213557; doi:10.1038/s41598-022-14596-x)
Supplement: Supplementary file 1 — Supplementary Information. [file 41598_2022_14596_MOESM1_ESM.docx]

Modeling and characterization of the electrical conductivity on metal nanoparticles/carbon nanotube/polymer composites

Supplementary Material

Fig S1 shows the SEM morphology of (a, b) Au/CNT, (c, d) Ni/CNT, (e, f) Cu/CNT and (g, h) AuCu/CNT.


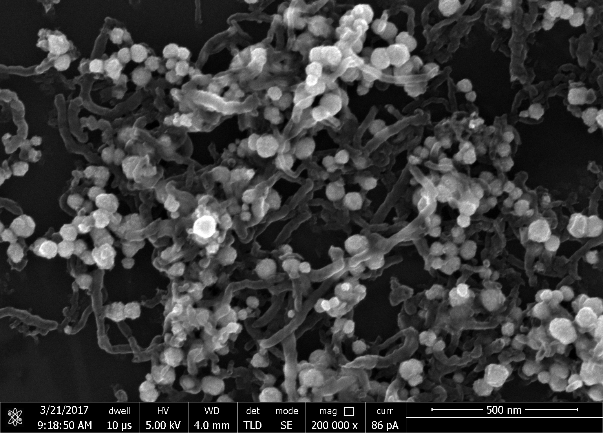

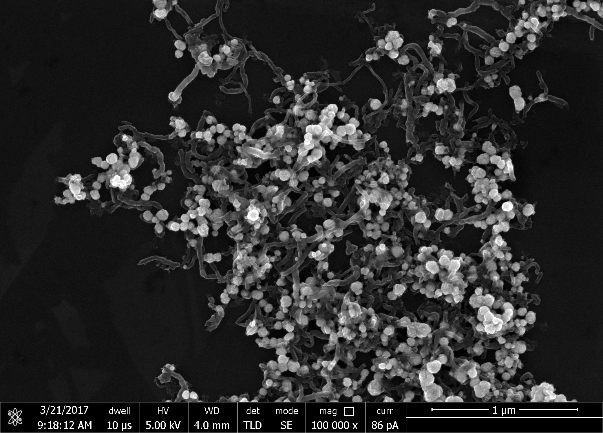


**e)**

**f)**


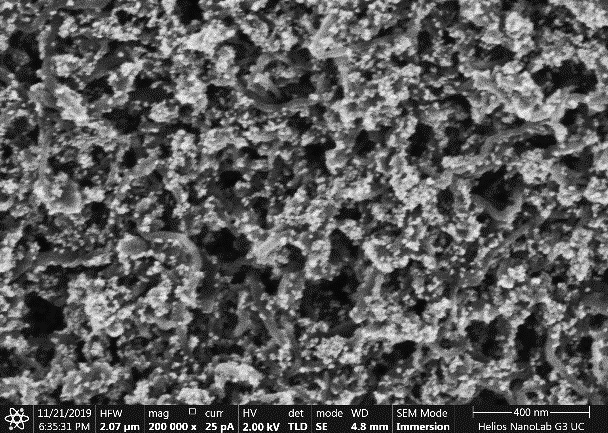

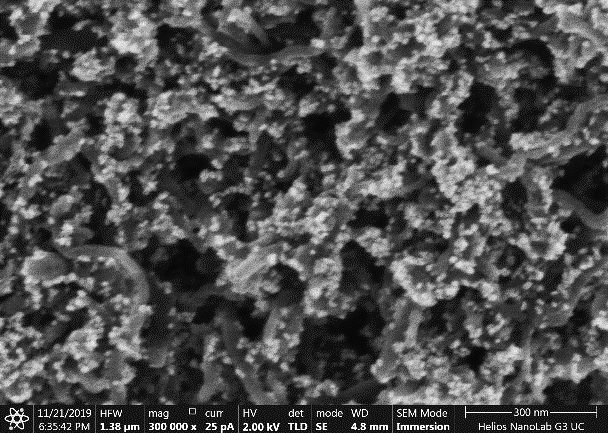

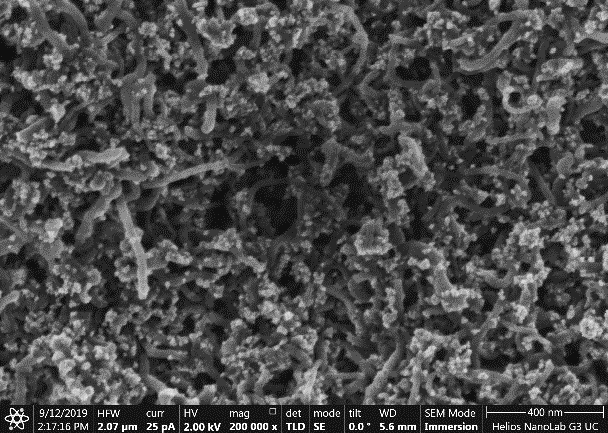

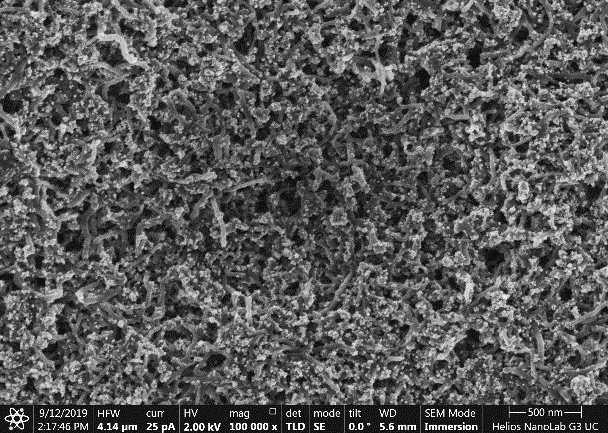


**a)**

**b)**

**c)**

**d)**


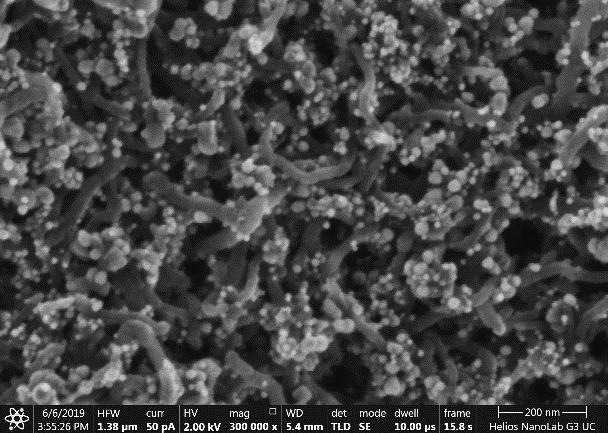

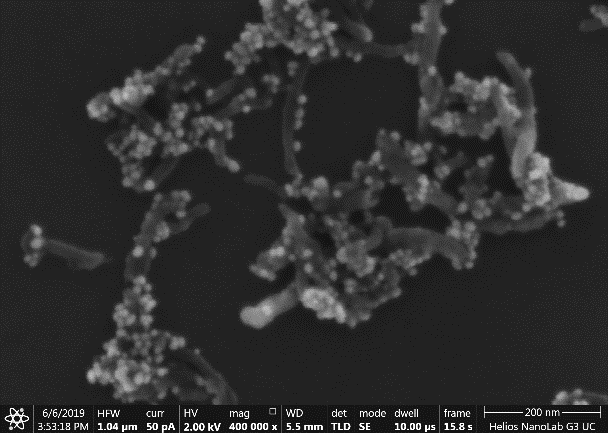


**g)**

**h)**

Figure S1. The SEM morphology of (a, b) Au/CNT, (c, d) Ni/CNT, (e, f) Cu/CNT, (g, h) AuCu/CNT.

Fig S2 shows the SEM morphology of (a, b) Ni/CNT/PDMS, (c, d) Au/CNT/PDMS, (e) Cu/CNT/PDMS and (f) AuCu/CNT/PDMS.


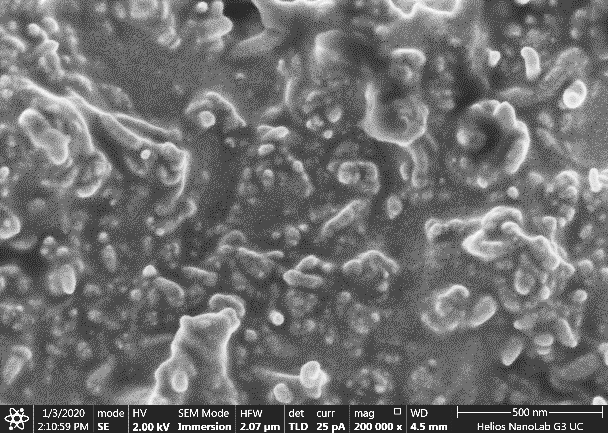

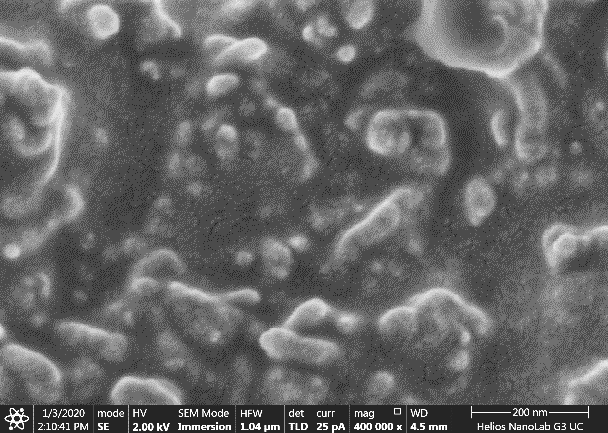

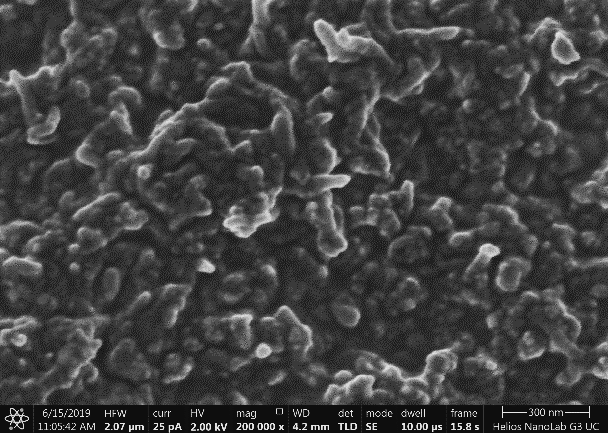

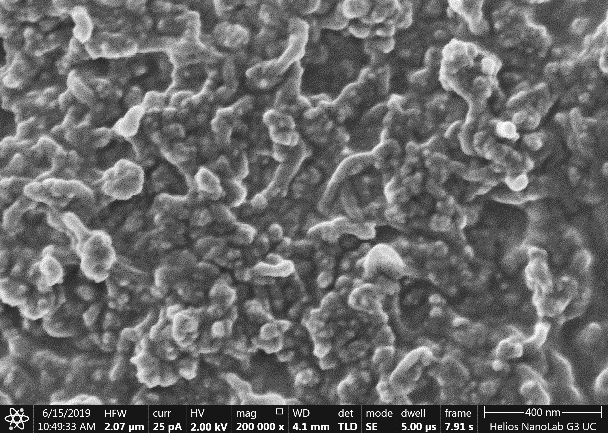

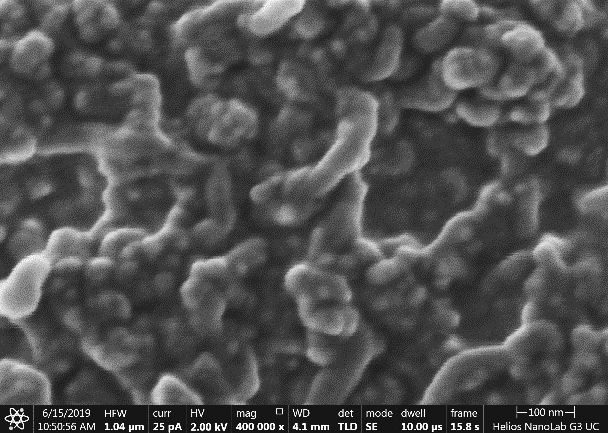

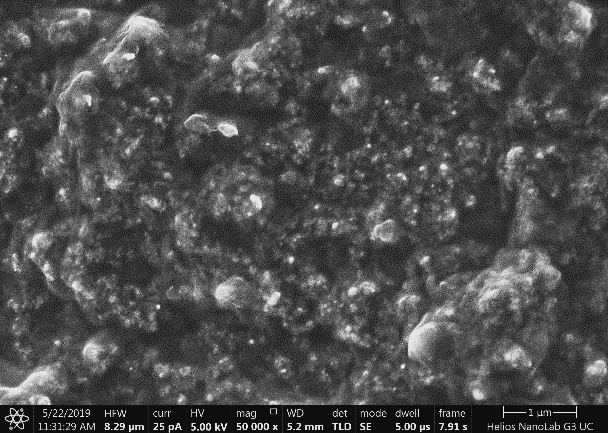


**a)**

**b)**

**c)**

**d)**

**e)**

**f)**

**Ni/CNT**

**Au/CNT**

**AuCu/CNT**

**Cu/CNT**

Figure S2. The SEM morphology of (a, b) Ni/CNT/PDMS, (c, d) Au/CNT/PDMS, (e) Cu/CNT/PDMS, (f) AuCu/CNT/PDMS.

Table 1. The specific speed information to fabricate the Metal/CNT/PDMS composite films.

| CNT loading | 2 wt.% | 4 wt.% | 6 wt.% | 8 wt.% |
| --- | --- | --- | --- | --- |
| CNT | 355rpm | 580rpm | 945rpm | 1520rpm |
| Ni/CNT | 400rpm | 630rpm | 980rpm | 1625rpm |
| Cu/CNT | 390rpm | 610rpm | 960rpm | 1570rpm |
| Au/CNT | 405rpm | 625rpm | 990rpm | 1670rpm |
| AuCu/CNT | 425rpm | 635rpm | 995rpm | 1695rpm |
